# Sodium Cuminate Inhibits the Mycelial Growth of *Penicillium digitatum* by Inducing Oxidative Stress and Damaging the Cell Membrane

**DOI:** 10.3390/jof11090612

**Published:** 2025-08-22

**Authors:** Mingchen Yang, Yonghua Zhang, Xiaoli Tan, Lu Li, Qiuli OuYang, Nengguo Tao

**Affiliations:** School of Chemical Engineering, Xiangtan University, Xiangtan 411105, China; 202021002081@smail.xtu.edu.cn (M.Y.); 202031000185@smail.xtu.edu.cn (Y.Z.); tanxiaoli@xtu.edu.cn (X.T.); lilu@xtu.edu.cn (L.L.)

**Keywords:** citrus, *Penicillium digitatum*, sodium cuminate, cell membrane, oxidative stress

## Abstract

Green mold formed by *Penicillium digitatum* is a major disease that limits the yield and overall value of postharvest citrus fruits. The antifungal activity of sodium cuminate (SC) against *P. digitatum* and the corresponding mechanism were explored in this research. The minimal inhibitory concentration (MIC) and minimal fungicidal concentration (MFC) of SC against *P. digitatum* were 0.4 and 0.8 g L^−1^, respectively. SC (8× MFC) reduced the incidence of disease in Ponkan fruits without compromising their quality. The results of CFW staining and extracellular alkaline phosphatase assays revealed that 1/2MIC SC for 30 min had no impact on the cell wall integrity of *P. digitatum*. In contrast, 1/2MIC SC apparently destroyed cell membrane integrity, as shown by the increase in the content of reactive oxygen species (ROS), malondialdehyde, and H_2_O_2_. The addition of exogenous cysteine (Cys) or diphenyleneiodonium chloride (DPI) significantly mitigated the cytotoxic effects of SC. At the same time, mitochondrial membrane potential was significantly decreased by 1/2MIC SC, and the addition of exogenous Cys or DPI restored it to normal levels. In summary, the antifungal capacity of SC might be attributable to membrane damage in *P. digitatum* caused by oxidative stress.

## 1. Introduction

Citrus fruits are among the most highly produced fruits in the world and have emerged as one of the most important crops. *Penicillium digitatum* is a pathogen that causes green mold, a major citrus disease, and limits the quantity and quality of postharvest citrus fruits globally [1]. Currently, the primary method for controlling postharvest citrus diseases is the application of chemical fungicides [2,3]. However, the widespread utilization of such chemical fungicides has created some major problems, including environmental contamination and decreased efficiency owing to pathogen resistance, posing risks to human health [4]. In light of these challenges, the development of eco-friendly alternatives has accelerated. Promising approaches include the use of antagonistic microorganisms (e.g., lactic acid bacteria, *Bacillus velezensis* strain S161, and yeasts), which have demonstrated protective and curative effects rivaling those of synthetic fungicides [5,6,7]. Similarly, plant-derived products, including plant extracts, essential oils, and bioactive compounds, exhibit dual functionality through direct antifungal activity and host defense induction, enabling their integration into disease management programs [4,8,9]. Additionally, edible antifungal coatings based on natural waxes or plant materials have emerged as safe technologies that simultaneously address physiological deterioration and pathological issues while enhancing fruit glossiness and extending shelf life [8,10,11]. Despite these advances, most alternative strategies have seen limited commercial adoption in the citrus industry due to scalability challenges and inconsistent real-world performance [7,8,9].

Recently, some safe organic acids and their salts have been explored as fungicides for postharvest disease control [8,11]. Cuminic acid (p-isopropyl benzoic acid, CA), belonging to the benzoic acid group, was isolated from the seeds of *Cuminum cyminum* L. and displayed a potent inhibitory effect on *Sclerotinia sclerotiorum* and *Phytophthora capsici* [12,13]. CA inhibited the mycelial growth of 54 *P. capsici* isolates by decreasing the levels of pyruvic acid and ATP and ATPase activity [14]. CA suppressed the mycelial growth of five *Fusarium oxysporum* and four *Colletotrichum lagenarium* strains in cucumber, with mean EC_50_ values of 0.02566 ± 0.00302 and 0.02953 ± 0.00318 g L^−1^, respectively [15]. Therefore, it can be safely applied to preserve fruits and vegetables and has the potential for development into a natural preservative. Because CA does not easily dissolve in water at room temperature [16], it is usually dissolved in a methanol solution to prepare a stock solution before use [13,14,15], greatly limiting its practical application in fruit and vegetable storage. An effective method to overcome this drawback is to obtain the corresponding salt via an acid–base neutralization reaction. Taking advantage of the high solubility of alkali metallic elements in water and polar solvents, Matejczyk et al. [17] used LiOH or NaOH to convert caffeic acid into caffeic acid salts with increased water solubility. Moreover, the caffeic acid salts integrating Li or Na had superior antimicrobial activity against *Candida* sp. than caffeic acid. Organic acid salts that are generally recognized as safe (GRAS) combine safety, solubility, and compatibility with ease of commercial handling to provide a viable means of controlling postharvest disease [3,8,10].

The objectives of this research were to (a) use an acid–base neutralization reaction to convert water-insoluble CA into water-soluble sodium cuminate (SC), (b) assess the effects of SC on *P. digitatum* both in vitro and in vivo, and (c) study the potential antifungal mechanism of SC against *P. digitatum*.

## 2. Materials and Methods

### 2.1. Fungal Strains and Citrus Fruits

*P. digitatum* (temporary lab code: PD), originally isolated from rotten citrus fruits and first characterized by Tao et al. [18], was maintained at 25 ± 2 °C after purification and morphological confirmation. The ITS sequence of strain PD was determined and deposited in GenBank under accession number PX128866.

The mature fruits of Ponkan (*Citrus reticulata* Blanco cv. Ponkan) with a uniform size and no scars were collected from a garden located in Xiangxi, China on December, 2022.

### 2.2. SC Preparation

CA (98%) was commercially obtained from Aladdin (Shanghai, China). SC was prepared according to the method reported by Matejczyk et al. [17]. CA was dissolved in a NaOH solution at a stoichiometric proportion of 1:1 to produce SC. The reaction products were further analyzed by the Fourier transform infrared (FT-IR) and HPLC spectra.

### 2.3. The Antifungal Activity of SC Against P. digitatum

The antifungal efficacy of SC against *P. digitatum* mycelial growth was measured using the agar dilution method [19]. The ultimate SC concentrations in PDA medium were 0, 0.20, 0.40, 0.80, and 1.60 g L^–1^. The lowest concentration that totally inhibited the *P. digitatum* growth after 2 days of incubation was designated as the minimal inhibitory concentration (MIC). The lowest dose that inhibited 99.5% of *P. digitatum* growth after 4 day of incubation was considered as the minimum fungicidal concentration (MFC). Every treatment was applied in three duplicates.

### 2.4. The Effects of SC on Disease Control and Quality of Citrus Fruit Inoculated with P. digitatum

A previously approved procedure was used to conduct the in vivo testing [19]. Fresh citrus fruits were cleaned with distilled water after 2 min of surface sterilization in a 2% sodium hypochlorite solution (*v*/*v*), pierced with a sterile scalpel to a depth and width of 3 mm, inoculated with 10^−6^ L of *P. digitatum* spore suspension (10^8^ spores L^−1^), and allowed to air dry. After inoculation, the treatments fruits were immersed in SC solutions (2×, 4×, and 8× MFC); the control group consisted of Pokan fruits that were bathed in sterile water, and those in the positive control were immersed in 0.025% prochloraz solution. All fruits were stored at 25 ± 2 °C and 85–90% relative humidity for 6 days. Ten Ponkan fruits were used in each experimental replication, and every treatment was in three duplicates.

Fruit samples were picked at random from each group after storage at an interval of 1 day to assess the following parameters: weight loss rates, coloring index, hardness, pH, total soluble solids (TSS) contents, titratable acidity (TA), and Vc contents [9]. Fruits treated with sterile water were used as the control.

### 2.5. Effects of SC on Naturally Occurring Diseases and Quality of Citrus Fruits

The Ponkan fruits were washed with tap water after being left for a day. After natural air drying, the fruits were immersed in 8× MFC SC or 0.025% prochloraz solution for 2 min. The fruits were stored in the natural environment after air drying, and the disease incidence and quality of the fruits were recorded for 0, 10, 20, and 30 days. All fruits were air-dried and stored in a natural environment. Each treatment group had 3 repetitions, with 150 fruits per repetition, and physiological indicators were evaluated in 3 replicates per group. The fruits treated with water were used as a negative control.

### 2.6. Effects of SC Treatment on the Cell Wall Integrity of P. digitatum

Various SC concentrations (0, 1/2 MIC, and MIC) in PDB were applied to *P. digitatum* mycelia that had been cultivated for 2 days for 0, 30, 60, and 120 min. Cell wall integrity was evaluated using calcofluor white (Sigma, St. Louis, MO, USA) staining in conjunction with ECLIPSE TS100 fluorescence microscopy (Nikon, Tokyo, Japan) [20].

Extracellular alkaline phosphatase (AKP) activity in *P. digitatum* mycelia was evaluated using the AKP kit (Nanjing Jiancheng, Nanjing, China) in accordance with the instructions. There were three repetitions of each experiment.

### 2.7. Effect of SC Treatment on the Cell Membrane Integrity of P. digitatum

Propidium iodide (PI) staining in conjunction with ECLIPSE TS100 fluorescence microscopy (Nikon, Tokyo, Japan) was employed to evaluate the plasma membrane integrity of *P. digitatum* mycelia cultivated with various SC concentrations (0, 1/2 MIC, and MIC) in PDB, as described previously [21]. ImageJ was used to analyze the fluorescence intensity of the images.

### 2.8. Effects of SC Treatment on Oxidative Stress in P. digitatum

The ROS assay kit (DCFH-DA) (Solarbio, Beijing, China) was employed to evaluate the ROS levels in *P. digitatum* mycelia cultivated with various SC concentrations (0, 1/2 MIC, and MIC) in PDB, as described previously. The fluorescence intensity of the images was analyzed using ImageJ software.

Malondialdehyde (MDA) and H_2_O_2_ contents of *P. digitatum* cells were evaluated using a spectrophotometer with commercial kits (Solarbio, Beijing, China) and in accordance with the instructions.

### 2.9. Effects of Exogenous Cysteine (Cys) or Diphenyleneiodonium Chloride (DPI) on ROS-Related Biochemical Parameters

The ROS in *P. digitatum* mycelia cultured with 0, 1/2 MIC SC, 1/2 MIC SC + 500 μM Cys (antioxidant cysteine), and 1/2 MIC SC + 1 μM diphenyleneiodonium chloride (DPI, a NADPH oxidase inhibitor) were evaluated using the JC-10 kit (Solarbio, Beijing, China).

GSH content and GST activity of *P. digitatum* cells treated with 0, 1/2 MIC SC, 1/2MIC SC + 500 μM Cys, and 1/2MIC SC + 1 μM DPI were determined with a spectrophotometer using the available kits (Solarbio, China) and in accordance with the instructions.

### 2.10. Effects of Exogenous Cys and DPI on the Surface Morphology of SC-Treated P. digitatum

The surface morphology of *P. digitatum* mycelia treated with 0, 1/2 MIC SC, 1/2 MIC SC + 500 μM Cys, and 1/2 MIC SC + 1 μM DPI for 30 min was examined using a JEM-1230 SEM (JEOL, Tokyo, Japan) [20].

### 2.11. Effects of Exogenous Cys and DPI on the Cell Membrane Integrity of SC-Treated P. digitatum

The cell membrane integrity of *P. digitatum* mycelia cultured with 0, 1/2 MIC SC, 1/2MIC SC + 500 μM Cys, and 1/2MIC SC + 1 μM DPI was assayed using PI staining.

The total lipid and ergosterol contents of *P. digitatum* treated with 0, 1/2 MIC SC, 1/2MIC SC + 500 μM Cys, and 1/2MIC SC + 1 μM DPI in PDB, as described above, were determined using the phosphovanillin method and HPLC method [21], respectively.

### 2.12. Effects of Exogenous Cys and DPI on the Mitochondrial Membrane Potential (MMP) of SC-Treated P. digitatum

The MMP of *P. digitatum* mycelia cultivated with 0, 1/2 MIC SC, 1/2MIC SC + 500 μM Cys, and 1/2MIC SC + 1 μM DPI was assayed using the JC-10 kit from Solarbio (Beijing, China).

### 2.13. Statistical Analyses

Data were evaluated using one-way ANOVA, followed by Duncan’s test, and presented as mean ± SD from three replicates. Statistical significance was evaluated at *p* < 0.05 using SPSS software version 16.0.

## 3. Results

### 3.1. The Antifungal Activity of SC Against P. digitatum

SC inhibited the development of *P. digitatum* mycelia in a dose-dependent manner (Table 1). When the concentration of SC exceeded 0.4 g L^−1^, *P. digitatum* mycelia cultured for 2 days showed little growth. When the treatment time increased to 4 days, 0.8 g L^−1^ SC completely inhibited mycelial growth. Therefore, the MIC and MFC values of SC against *P. digitatum* were 0.40 and 0.80 g L^−1^, respectively.

### 3.2. The Effect of SC on Green Mold in Citrus Fruit Incubated with P. digitatum

**Figure 1:**
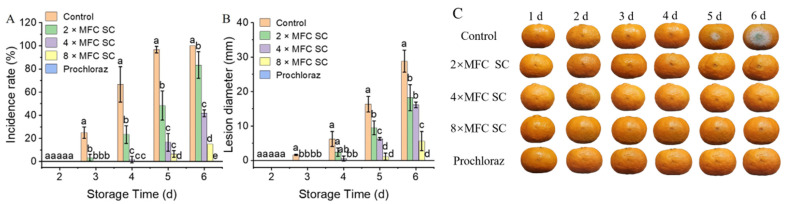
The effects of SC (0×, 2×, 4× and 8× MFC) and prochloraz on green mold incidence and fruit quality of Ponkan fruits inoculated with *P. digitatum*. (**A**) Disease incidence; (**B**) Lesion diameter; (**C**) Fruit appearance. The data presented are the means of pooled data. Error bars indicate the SDs of the means (*n* = 3). “a–e” indicates the difference among different treatment groups (*p* < 0.05).

The SC (2×, 4×, and 8× MFC) treatments not only delayed but also prolonged the onset of citrus green mold in citrus fruits. SC hindered the growth of *P. digitatum*, and only water spots showed an increase in lesion diameter in diseased areas, while the formation of mildew spots was delayed (Figure 1A–C). Slight water spots appeared on the surface of citrus fruits at the early stage of green mold growth. After 3 days, the fruits in the control and 2× MFC SC groups started to rot and showed slight water spots, with incidence rates of 15.00 ± 5.00% and 3.33 ± 2.89%, respectively, while the other treatment groups were not diseased. The fruits in the 4× and 8× MFC SC treatment groups started to show signs of infection after 4 days and 5 days of storage, with occurrence rates of 16.66 ± 7.63% and 6.66 ± 2.88%, respectively, considerably lower than that in the control group (93.33 ± 5.77%, *p* < 0.05). After 6 days of storage, every fruit in the control group decayed, with obvious green mold spots, whereas the occurrence rate in the 8× MFC SC treatment group was only 15.00 ± 0.00% with a small number of water spots, and the fruits in the prochloraz treatment group remained healthy.

After 5 days of storage, the water loss rates of SC-treated Ponkan fruits were dramatically reduced compared to the control group. The water loss rates in the 2×, 4×, and 8× MFC SC treatments and prochloraz treatment were 3.85 ± 0.52, 3.45 ± 0.55, 3.29 ± 0.83, and 3.77 ± 0.47%, respectively, which were significantly lower than that in the control group (4.74 ± 0.57%, *p* < 0.05) (Table 2). In addition, the Vc content in each group maintained an upward trend during storage. On day 5 of storage, the Vc contents in the 2×, 4×, and 8× MFC SC treatments and prochloraz treatment were 36.36 ± 2.90, 38.53 ± 3.63, 37.59 ± 0.85, and 35.72 ± 1.02 mg 100 g^−1^ respectively, which were significantly higher than that in the control group (31.18 ± 2.53 mg 100 g^−1^, *p* < 0.05), indicating that SC treatment could stimulate the accumulation of Vc content in Ponkan fruits. On the first day of storage, the TSS contents in the control group and the 2× MFC SC treatment group were 14.84 ± 0.40 and 15.02 ± 0.37%, respectively, which were significantly lower than those in the other groups (*p* < 0.05). With prolonged storage time, the TSS contents in each treatment group did not change significantly. In addition, SC treatment did not affect the fruit coloring index, pH, TA, or hardness during the entire storage period.

### 3.3. Effects of SC Treatment on Naturally Occurring Diseases in Citrus Fruits

**Figure 2:**
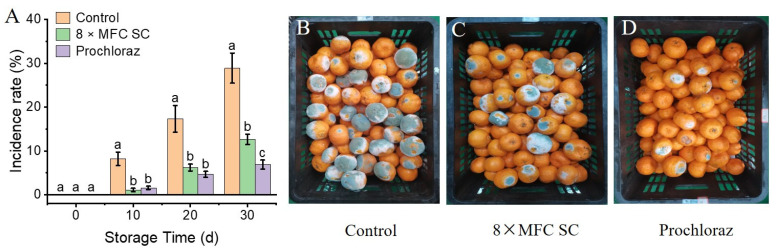
The effects of SC (0×, and 8× MFC) and prochloraz on naturally occurred diseases incidence and fruit quality of Ponkan fruits. (**A**) Fruit appearance; (**B**–**D**) Fruit appearance after 30 days incubation. The data presented are the means of pooled data. Error bars indicate the SDs of the means (*n* = 3). “a–c” indicates the difference among different treatment groups (*p* < 0.05).

As shown in Figure 2A–D, Ponkan fruits in all groups began to rot at 10 days of storage, but the SC- and prochloraz-treated fruits had significantly lower disease incidence rates (1.11 ± 0.38% and 1.56 ± 0.38%, respectively) than the control group (8.22 ± 1.54%, *p* < 0.05). The disease incidence rose considerably in all groups as the storage duration progressed. After 30 days of storage, Ponkan fruits in the control group had a considerably greater incidence of disease (28.89 ± 3.42%) compared to the SC and prochloraz treatments (12.67 ± 1.15% and 6.89 ± 1.02%, respectively) (*p* < 0.05).

The effects of 8× MFC SC treatment on the quality of naturally infected Ponkan fruits are shown in Table 3. Throughout the storage period, the coloring index, pH, TSS, and TA contents in each SC-treated group remained similar to those in the control group. However, SC effectively reduced the water loss rates of Ponkan fruits. The water loss rates for the SC- and prochloraz-treated groups were 3.41 ± 0.83% and 3.23 ± 1.01%, respectively, at 30 days of storage. They were considerably lower than that of the control group (5.10 ± 0.81%, *p* < 0.05). Similarly, the hardness of the SC-treated fruits remained at a high level during storage. At 30 days, the hardness of the SC-treated fruits was 5.58 ± 0.07 N, which is significantly higher than that of the control group (5.10 ± 0.06 N, *p* < 0.05). At the same time, after 30 days of storage, the Vc content in the SC-treated group (32.35 ± 0.71 mg 100 g^−1^) was significantly higher than that in the control group (27.60 ± 0.86 mg 100 g^−1^) and the prochloraz-treated group (30.62 ± 0.59 mg 100 g^−1^).

### 3.4. SC Does Not Compromise the Cell Wall Integrity of P. digitatum

**Figure 3:**
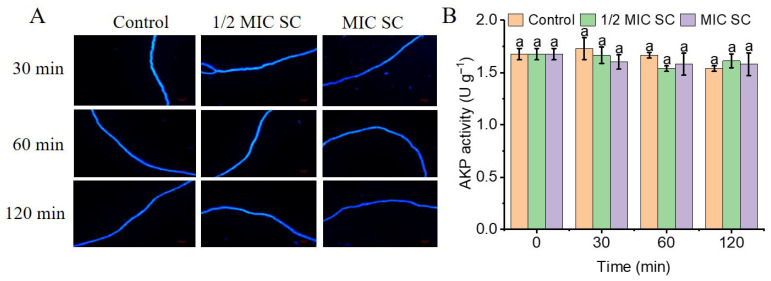
The effects of SC on the cell walls of *P. digitatum*. (**A**) The cell wall integrity of *P. digitatum*; (**B**) The extracellular AKP activity of *P. digitatum*. The data presented are the means of pooled data. Error bars indicate the SDs of the means (*n* = 3). “a” indicated that there was no difference between different treatment groups (*p* < 0.05).

The increase in SC concentration and treatment time did not affect the blue fluorescence of *P. digitatum* mycelia, and the septa were clearly visible, with no noticeable brightness difference from the control group, demonstrating that the cell wall remained intact (Figure 3A). SC was not harmful to the cell wall integrity of *P. digitatum*, as evidenced by the extracellular AKP activity in the SC-treated groups being comparable to that in control samples (Figure 3B).

### 3.5. SC Treatment Impairs the Cell Membrane Integrity of P. digitatum

**Figure 4:**
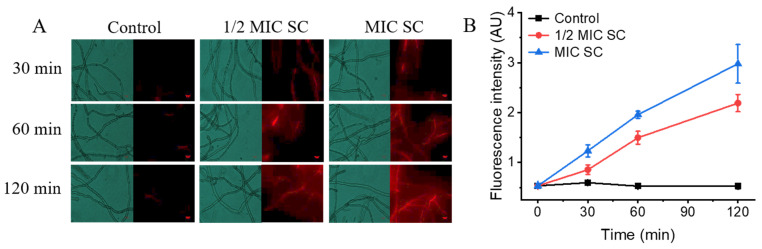
The effects of SC on the cell membrane integrity of *P. digitatum*. (**A**) The cell membrane integrity of *P. digitatum*; (**B**) The mycelia fluorescence times. The data presented are the means of pooled data. Error bars indicate the SDs of the means (*n* = 3).

The PI staining results in the control group show no fluorescence or weak red fluorescence within 120 min (Figure 4A). However, the 1/2MIC- and MIC SC-treated groups showed obvious red fluorescence at 30 min, with fluorescence intensity values 1.44- and 2.08-fold higher than that of the control group, respectively. With the progression of treatment time, the fluorescence values of the 1/2MIC and MIC SC treatment groups peaked at 120 min and were 4.21 and 5.68 times that in the control group (Figure 4B), indicating that SC could extensively disrupt the cell membrane of *P. digitatum*.

### 3.6. SC Induces a Massive Accumulation of ROS and Lipid Peroxidation in P. digitatum

**Figure 5:**
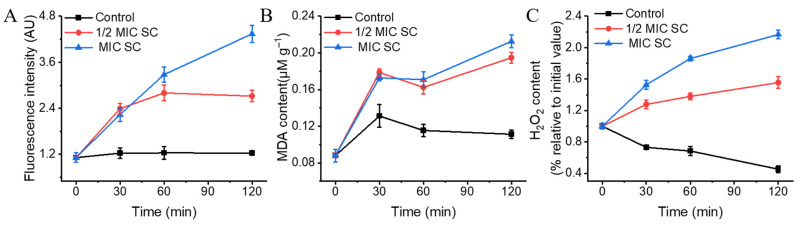
The effects of SC on the ROS contents and lipid peroxidation of *P. digitatum*. (**A**) The mycelia fluorescence times in the ROS accumulation of *P. digitatum*; (**B**) The MDA content; (**C**) The H_2_O_2_ content. The data presented are the means of pooled data. Error bars indicate the SDs of the means (*n* = 3).

At 120 min of exposure, the green fluorescence was the brightest, and the fluorescence values of the 1/2MIC- and MIC SC-treated groups were 2.20 and 3.51 times higher than that of the control group (*p* < 0.05) (Figure 5A), suggesting the massive accumulation of ROS, which can ultimately damage the cell membrane of *P. digitatum* mycelia. Similarly, the H_2_O_2_ levels were also significantly increased by SC (Figure 5C). After 120 min of treatment, the H_2_O_2_ levels in the 1/2 MIC and MIC SC treatment groups increased by 70.19% and 171.35% compared to the control group, which was consistent with the change trend in ROS content.

The MDA content in the control group remained practically constant, whereas those in the SC treatment groups followed an overall upward trend (Figure 5B). After 120 min of treatment, the MDA contents in the 1/2 MIC- and MIC SC-treated groups were 0.195 ± 0.006 and 0.213 ± 0.007 mM g^−1^, respectively. These values were significantly higher than that in the control group (0.111 ± 0.005 mM g^−1^, *p* < 0.05), suggesting the occurrence of membrane lipid peroxidation.

### 3.7. The Addition of Exogenous Cys and DPI Alleviates the Oxidative Stress Caused by SC

**Figure 6:**
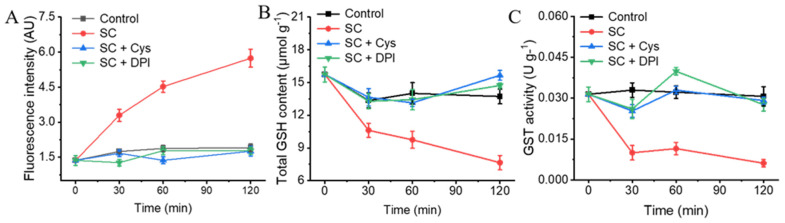
The effects of SC on the ROS and GSH content and GST activity of *P. digitatum* mycelia by addition of exogenous Cys and DPI. (**A**) The mycelia fluorescence times in the ROS accumulation of *P. digitatum*; (**B**) The GSH content; (**C**) The GST activity. The data presented are the means of pooled data. Error bars indicate the SDs of the means (*n* = 3).

To confirm whether the oxidative stress was induced by the SC treatment, two antioxidant substances, Cys and DPI, were added. The addition of exogenous Cys and DPI resulted in equivalent ROS levels in the control samples (Figure 6A). Following a 120 min treatment period, the ROS levels in the groups treated with exogenous Cys and DPI were 0.92- and 0.93-fold that in the control group, respectively.

The total GSH content in the control group remained constant for 120 min after treatment with SC, while the total GSH content in the 1/2MIC SC treatment group began to decrease significantly at 30 min (Figure 6B). After treatment for 30 min and 120 min, the GSH content in the 1/2MIC SC treatment group decreased by 30.18% and 55.00%, respectively, compared with the control group (*p* < 0.05). The total GSH content in the treatment group with exogenous Cys and DPI recovered to levels comparable to those in the control group. The total GSH content and GST activity follow comparable change trends. The GST activity in the 1/2MIC SC treatment group was still significantly lower than that in the control group (*p* < 0.05) (Figure 6C). At 120 min, the GST activity in the 1/2MIC SC treatment group was only 19.99% of that in the control group. After adding Cys and DPI, the GST enzyme activity in *P. digitatum* recovered to the same level as that of the control group, indicating that SC treatment led to oxidative stress in the cells and that this adverse effect could be mitigated by the addition of exogenous Cys and DPI.

### 3.8. The Addition of Exogenous Cys and DPI Mitigates the Damage to Surface Morphology Caused by SC

**Figure 7:**
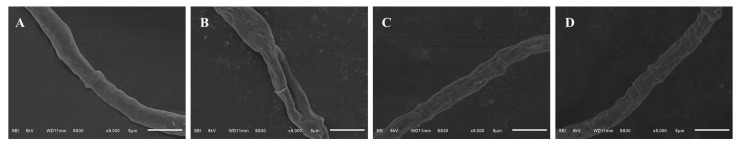
The effects of SC on the surface morphology of *P. digitatum* mycelia by addition of exogenous Cys and DPI. (**A**) Untreated control; (**B**) SC; (**C**) SC + Cys; (**D**) SC + DPI.

The addition of exogenous Cys and DPI effectively reduced the degree of damage caused by SC to *P. digitatum* mycelia (Figure 7). After treatment for 30 min, the mycelia in the 1/2 MIC SC treatment group showed a large area of depression (Figure 7B). However, the mycelia reverted to their former full and smooth state, comparable to the control group, after the addition of exogenous Cys and DPI (Figure 7A,C,D).

### 3.9. The Addition of Exogenous Cys and DPI Mitigates the Damage to Cell Membrane Integrity and Lipid Peroxidation Caused by SC

**Figure 8:**
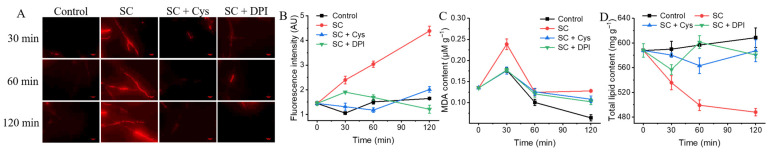
The effects of SC on the cell membrane integrity and lipid peroxidation of *P. digitatum* mycelia by addition of exogenous Cys and DPI. (**A**) The cell membrane integrity of *P. digitatum*; (**B**) The mycelia fluorescence times; (**C**) The MDA content; (**D**) The total lipid content. The data presented are the means of pooled data. Error bars indicate the SDs of the means (*n* = 3).

The cell membrane integrity of *P. digitatum* was severely disrupted by SC treatment. The addition of exogenous Cys and DPI mitigated the damage that SC caused to the cell membrane (Figure 8A,B). At 30 min and 120 min, the fluorescence intensities of mycelia treated with 1/2MIC SC were 2.27 and 4.17 times higher than that in the control group, respectively. In contrast, the fluorescence intensity decreased to the same level as that in the control group after the addition of exogenous Cys and DPI.

As the treatment period progressed, the MDA contents in the 1/2MIC SC treatment group increased to considerably greater levels than those in the control group (Figure 8C). At 30 min, the MDA content peaked at 1.35 times that in the control group. After the addition of exogenous Cys and DPI, the MDA content decreased to a level close to that in the control group.

SC considerably lowered the total lipid contents of *P. digitatum* (Figure 8D). For 120 min, the total lipid contents in the control group remained relatively constant; however, at 30 min, they significantly decreased in the 1/2MIC SC treatment group, and at 120 min, they were 80.17% of those in the control group (*p* < 0.05). After the addition of exogenous Cys and DPI, the total lipid contents were similar to those in the control group. These findings demonstrate that the addition of exogenous Cys and DPI effectively lessens the impact of SC on membrane lipid peroxidation in *P. digitatum*.

### 3.10. The Addition of Exogenous Cys and DPI Mitigates the Decrease in MMP Caused by SC

**Figure 9:**
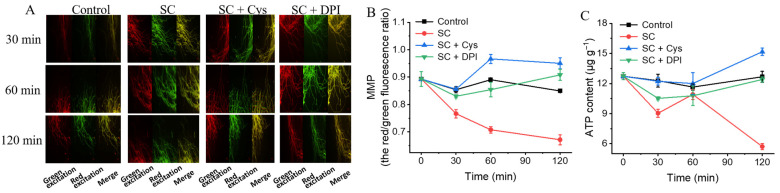
The effects of SC on the MMP and ATP content of *P. digitatum* mycelia by addition of exogenous Cys and DPI. (**A**) The MMP of *P. digitatum*; (**B**) The mycelia red/green fluorescence ratio; (**C**) The ATP content. The data presented are the means of pooled data. Error bars indicate the SDs of the means (*n* = 3).

The MMP in the SC treatment group dropped dramatically to 0.76 ± 0.02 at 30 min (Figure 9A,B, *p* < 0.05) over the treatment period, whereas the MMP in the control group remained relatively constant, suggesting that normal mitochondrial function was disrupted. After the addition of exogenous Cys and DPI, the MMP returned to normal levels. After 120 min of treatment, the MMPs in the CS + Cys and CS + DPI treatment groups were 1.12 and 1.07 times higher than that in the control group, indicating that Cys and DPI delayed the decrease in MMP caused by SC treatment.

## 4. Discussion

Some organic acid salts are effective at inhibiting common pathogens and controlling the rates of rot caused by fruit and vegetable diseases [8,22,23,24]. Sodium benzoate and potassium sorbate demonstrate efficacy against key citrus molds, including *P. digitatum*, but their practical utility is limited; this is particularly exemplified by potassium sorbate, which severely compromises protective wax coatings, exacerbating fruit weight loss by up to 65% compared to wax-only treatments [25,26]. In this study, SC emerges as a distinct strategic alternative, exhibiting potent inhibition of *P. digitatum* at MIC and MFC values of 0.4 and 0.8 g L^−1^, respectively, alongside quality-preserving attributes. At the 8× MFC concentration, SC significantly reduced disease incidence and lesion diameter in both artificially inoculated and naturally infected citrus fruits; concurrently, it mitigated weight loss by reducing fruit water loss and respiratory consumption [27]; maintained peel hardness, possibly through suppression of cell-wall-degrading enzymes [28]; and enhanced Vc accumulation during storage. Crucially, SC’s performance contrasts with that of conventional organic salt applications, where antifungal efficacy often comes at the expense of fruit quality. Additionally, SC aqueous solution can be easily included in current packing line techniques—unlike antagonistic microorganisms that need specialist equipment and non-water-soluble plant extracts that need to be emulsified—and can reduce commercial usage costs. As a result, SC is a viable candidate for implementation within industrial frameworks requiring both decay management and quality preservation. However, industrial scalability needs to be further validated under commercial conditions, particularly in terms of efficacy against mixed pathogen infections and stability in high-humidity environments.

Many antifungal drugs target cell walls and membranes, which play crucial roles in signal transduction, material transport, and energy conversion [29,30,31]. Cuminic acid treatment enhanced the electrolyte leakage and cell membrane permeability of *P. capsici* mycelia, resulting in damage to the cell membrane [14]. Na_2_SiO_3_ and EDTA-Na_2_ disrupted the mycelial cell membranes of postharvest citrus pathogens, which resulted in significant leakage of nucleic acids [3]. In this investigation, SC exhibited no impact on the cell wall integrity of *P. digitatum* but did damage the cell membrane integrity at 30 min, indicating that cell membrane damage is the main mechanism through which SC suppresses the growth of *P. digitatum*. ROS buildup is typically linked to cell membrane damage, which can cause oxidative stress, damage biomacromolecules, and ultimately result in cell death [11,21]. According to Shu et al. [32], ε-polylysine induced the formation and accumulation of endogenous ROS and caused severe lipid peroxidation, resulting in cell membrane damage. 6-Pentyl-2H-pyran-2-one compromised cell membrane integrity of *Clarireedia jacksonii*, and a marked increase in ROS and MDA levels was observed [33]. MDA is a significant byproduct used to analyze the process of lipid peroxidation [34]; it can react with proteins and nucleic acids, leading to cross-linking polymerization between molecules, further aggravating cell damage and eventually leading to a decrease in cell membrane fluidity, an increase in cell membrane permeability, and changes in cell structures and functions [21]. Following a 30 min SC treatment, the contents of ROS, H_2_O_2_, and MDA in *P. digitatum* cells increased sharply. Lipid peroxidation brought on by a significant buildup of ROS is thought to be the cause of SC-induced damage to the cell membrane of *P. digitatum*. In addition, lipid peroxidation has been demonstrated to be able to induce oxidative stress and damage biomacromolecules in *Alternaria alternata*, *R. solani*, and *Escherichia coli*, ultimately leading to cell death [32,35,36]. Therefore, it is speculated that SC’s mode of action may be to induce an increase in H_2_O_2_, which leads to oxidative stress, lipid peroxidation of the cell membrane, and degradation of the cell membrane structure, ultimately affecting the normal growth of mycelia [36].

The addition of Cys and DPI will increase glutathione synthesis or promote an increase in antioxidant enzyme activities, thus eliminating excessive ROS [37,38]. MMP, a crucial biomarker of mitochondrial activity, reflects cell health, and its reduction is a signal of cell autophagy and apoptosis [39]. Excessive ROS reduce MMP and respiratory chain activities, leading to mitochondrial dysfunction [40]. Kwun and Lee [41] demonstrated that quercetin induced yeast apoptosis through the generation of ROS, depolarization of the mitochondrial membrane, and disturbance of the whole antioxidant system. In this investigation, as compared to the control, SC reduced MMP and intracellular ATP content. After the addition of exogenous Cys and DPI, the ROS content and oxidative damage caused by SC were effectively reduced, so the MMP was restored to normal levels, and the balance of intracellular energy substances and the balance between GSH and GST were maintained. Plačková et al. [42] found that 9-norbornyl-6-chloropurine decreased the GSH content in cells and caused ROS accumulation and lipid peroxidation, which led to the loss of MMP, but it was restored to normal levels after adding a ROS scavenger. Additionally, these findings align with those of Han et al. [43] and Liu et al. [44], who reported that the addition of exogenous Cys mitigated the effects of sodium dehydroacetate on oxidative damage, and that the addition of exogenous DPI can inhibit patulin-induced cytotoxicity and apoptosis, reduce oxidative damage, and maintain MMP and ATP content. Meanwhile, exogenous cysteine and DPI also significantly reduced the cell membrane damage resulting from SC treatment: the contents of total lipids and MDA were roughly comparable to those in the control group, and cells maintained normal cell morphology, further demonstrating the connection between oxidative-stress-induced cell membrane damage and the antifungal mechanism of SC.

## 5. Conclusions

SC can be applied as an alternative strategy for managing green mold affecting citrus fruits after harvest. In conclusion, our physiological and cytological assays indicate that SC inhibits *P. digitatum* growth by disrupting mitochondrial function, depleting ATP content, inducing ROS accumulation, and compromising cell membrane integrity. While these observations consistently align with an oxidative-damage-mediated antifungal mechanism, further transcriptomic or proteomic investigations are warranted to elucidate the precise molecular pathways governing these effects.

## Figures and Tables

**Table 1:** SC inhibited the growth of *P. digitatum* in vitro.

SC (g L^−1^)	Inhibitory Rate (%)			
	1 Day	2 Day	3 Day	4 Day
0.2	100.00 ± 0.00 a	81.25 ± 0.00 a	78.23 ± 2.42 b	70.29 ± 3.69 c
0.4	100.00 ± 0.00 a	100.00 ± 0.00 a	100.00 ± 0.00 a	92.57 ± 2.62 b
0.8	100.00 ± 0.00 a	100.00 ± 0.00 a	100.00 ± 0.00 a	100.00 ± 0.00 a
1.6	100.00 ± 0.00 a	100.00 ± 0.00 a	100.00 ± 0.00 a	100.00 ± 0.00 a

Note: Data presented are the means ± standard error of pooled data (*n* = 3). “a–c” indicates the difference among different treatment groups with the same storage time (*p* < 0.05).

**Table 2:** Effect of SC on the quality of citrus fruits inoculated with *P. digitatum.*

Physiological Indicators	Treatments	0 Day	1 Day	3 Day	5 Day
Weight loss (%)	Control	0.00 ± 0.00 a	1.59 ± 0.27 a	3.11 ± 0.43 a	4.74 ± 0.57 a
	Prochloraz	0.00 ± 0.00 a	1.18 ± 0.31 b	2.49 ± 0.26 a	3.77 ± 0.47 b
	2× MFC SC	0.00 ± 0.00 a	1.21 ± 0.29 b	2.58 ± 0.43 a	3.85 ± 0.52 b
	4× MFC SC	0.00 ± 0.00 a	1.10 ± 0.16 b	2.55 ± 0.40 a	3.45 ± 0.55 b
	8× MFC SC	0.00 ± 0.00 a	1.08 ± 0.24 b	2.01 ± 0.66 a	3.29 ± 0.83 b
Coloration index	Control	5.29 ± 0.40 a	5.52 ± 0.41 a	5.77 ± 0.44 a	6.08 ± 0.43 a
	Prochloraz	5.15 ± 0.31 a	5.31 ± 0.24 a	5.40 ± 0.21 a	5.69 ± 0.27 a
	2× MFC SC	5.17 ± 0.41 a	5.35 ± 0.43 a	5.58 ± 0.43 a	5.82 ± 0.40 a
	4× MFC SC	5.34 ± 0.45 a	5.64 ± 0.44 a	5.85 ± 0.47 a	6.06 ± 0.39 a
	8× MFC SC	5.28 ± 0.69 a	5.64 ± 0.64 a	5.81 ± 0.63 a	5.91 ± 0.69 a
pH	Control	2.63 ± 0.03 a	2.93 ± 0.00 a	2.72 ± 0.01 a	2.78 ± 0.01 a
	Prochloraz	2.63 ± 0.03 a	2.75 ± 0.01 a	2.72 ± 0.02 a	2.76 ± 0.02 a
	2× MFC SC	2.63 ± 0.03 a	2.79 ± 0.01 a	2.75 ± 0.01 a	2.83 ± 0.01 a
	4× MFC SC	2.63 ± 0.03 a	2.71 ± 0.03 a	2.78 ± 0.01 a	2.77 ± 0.02 a
	8× MFC SC	2.63 ± 0.03 a	2.71 ± 0.01 a	2.76 ± 0.01 a	2.77 ± 0.01 a
TSS (%)	Control	15.94 ± 0.28 a	14.84 ± 0.40 b	15.70 ± 0.35 a	16.38 ± 0.47 a
	Prochloraz	15.94 ± 0.28 a	17.24 ± 0.30 a	16.90 ± 0.54 a	16.79 ± 0.09 a
	2× MFC SC	15.94 ± 0.28 a	15.02 ± 0.37 b	15.96 ± 0.99 a	16.40 ± 0.22 a
	4× MFC SC	15.94 ± 0.28 a	16.60 ± 0.61 a	16.02 ± 0.28 a	16.23 ± 0.38 a
	8× MFC SC	15.94 ± 0.28 a	16.48 ± 0.31 a	16.77 ± 1.00 a	16.72 ± 0.50 a
TA (%)	Control	1.44 ± 0.18 a	1.03 ± 0.08 b	1.66 ± 0.23 a	1.45 ± 0.10 a
	Prochloraz	1.44 ± 0.18 a	1.47 ± 0.08 a	1.48 ± 0.13 a	1.63 ± 0.12 a
	2× MFC SC	1.44 ± 0.18 a	1.33 ± 0.06 a	1.71 ± 0.28 a	1.47 ± 0.17 a
	4× MFC SC	1.44 ± 0.18 a	1.56 ± 0.29 a	1.33 ± 0.18 a	1.44 ± 0.10 a
	8× MFC SC	1.44 ± 0.18 a	1.48 ± 0.05 a	1.56 ± 0.12 a	1.61 ± 0.13 a
Vc content (mg 100 g^−1^)	Control	27.32 ± 1.52 a	39.28 ± 1.29 a	28.79 ± 2.82 c	31.18 ± 2.53 b
	Prochloraz	27.32 ± 1.52 a	33.44 ± 0.99 b c	37.30 ± 1.02 a b	35.72 ± 1.02 a b
	2× MFC SC	27.32 ± 1.52 a	37.77 ± 3.69 a b	35.42 ± 1.18 a b	36.36 ± 2.90 a
	4× MFC SC	27.32 ± 1.52 a	35.33 ± 1.23 b c	33.63 ± 1.23 b	38.53 ± 3.63 a
	8× MFC SC	27.32 ± 1.52 a	31.28 ± 2.67 c	39.00 ± 2.31 a	37.59 ± 0.85 a
Hardness (N)	Control	5.21 ± 0.27 a	6.07 ± 0.07 a	5.88 ± 0.14 a	5.05 ± 0.19 b
	Prochloraz	5.21 ± 0.27 a	5.92 ± 0.12 a	5.97 ± 0.11 a	5.97 ± 0.09 a
	2× MFC SC	5.21 ± 0.27 a	6.00 ± 0.15 a	5.97 ± 0.08 a	5.91 ± 0.12 a
	4× MFC SC	5.21 ± 0.27 a	6.14 ± 0.09 a	6.01 ± 0.07 a	5.93 ± 0.10 a
	8× MFC SC	5.21 ± 0.27 a	5.92 ± 0.04 a	6.06 ± 0.10 a	6.00 ± 0.09 a

Note: Data presented are the means ± standard error of pooled data (*n* = 3). “a–c” indicates the difference among different treatment groups (*p* < 0.05).

**Table 3:** Effect of SC on the quality of naturally occurring citrus fruits.

Physiological Indicators	Treatments	0 Day	10 Day	20 Day	30 Day
Weight loss rate (%)	Control	0.00 ± 0.00 a	2.78 ± 0.63 c	3.76 ± 0.61 a	5.10 ± 0.81 a
	Prochloraz	0.00 ± 0.00 a	1.08 ± 0.69 a	2.38 ± 1.19 b	3.23 ± 1.01 b
	8× MFC SC	0.00 ± 0.00 a	1.76 ± 0.71 a b	2.53 ± 0.96 b	3.41 ± 0.83 b
Coloration index	Control	5.10 ± 0.57 a	5.85 ± 0.81 a	6.39 ± 0.53 a	6.67 ± 0.47 a
	Prochloraz	4.46 ± 0.57 a	5.31 ± 0.41 a	6.09 ± 0.32 a	6.44 ± 0.38 a
	8× MFC SC	4.88 ± 0.81 a	5.60 ± 0.67 a	6.19 ± 0.48 a	6.56 ± 0.30 a
pH	Control	2.63 ± 0.03 a	2.73 ± 0.01 a	2.81 ± 0.01 a	2.89 ± 0.01 a
	Prochloraz	2.63 ± 0.03 a	2.83 ± 0.01 a	2.83 ± 0.07 a	2.95 ± 0.01 a
	8× MFC SC	2.63 ± 0.03 a	2.64 ± 0.01 a	2.86 ± 0.02 a	2.99 ± 0.01 a
TSS (%)	Control	15.94 ± 0.28 a	16.68 ± 0.49 a	15.61 ± 0.13 a	16.46 ± 0.48 a
	Prochloraz	15.94 ± 0.28 a	15.48 ± 0.29 a	15.72 ± 0.13 a	16.67 ± 0.17 a
	8× MFC SC	15.94 ± 0.28 a	16.43 ± 0.30 a	15.93 ± 0.15 a	16.84 ± 0.18 a
TA (%)	Control	1.14 ± 0.23 a	1.41 ± 0.08 a	1.26 ± 0.05 a	1.40 ± 0.08 a
	Prochloraz	1.14 ± 0.23 a	1.47 ± 0.08 a	1.28 ± 0.06 a	1.37 ± 0.05 a
	8× MFC SC	1.14 ± 0.23 a	1.66 ± 0.12 a	1.41 ± 0.08 a	1.44 ± 0.11 a
Vc content (mg 100 g^−1^)	Control	27.32 ± 1.52 a	33.91 ± 2.83 a	27.60 ± 0.86 c	29.58 ± 0.16 b
	Prochloraz	27.32 ± 1.52 a	31.18 ± 2.08 a	30.62 ± 0.59 b	31.46 ± 0.16 a b
	8× MFC SC	27.32 ± 1.52 a	33.53 ± 1.33 a	32.35 ± 0.71 a	33.63 ± 2.47 a
Hardness (N)	Control	5.21 ± 0.27 a	5.46 ± 0.37 a	5.11 ± 0.07 b	5.10 ± 0.06 b
	Prochloraz	5.21 ± 0.27 a	5.91 ± 0.06 a	5.66 ± 0.19 a	5.63 ± 0.05 a
	8× MFC SC	5.21 ± 0.27 a	5.70 ± 0.05 a	5.54 ± 0.15 a	5.58 ± 0.07 a

Note: Data presented are the means ± standard error of pooled data (*n* = 3). “a–c” indicates the difference among different treatment groups (*p* < 0.05).

## Data Availability

The datasets generated during and/or analyzed during the current study are available from the corresponding author upon reasonable request.
